# Fixed-Candidate Reliability Auditing for Closed-Set Binary Function Retrieval Under Known-Source Cross-Compilation Protocols

**DOI:** 10.3390/e28080938

**Published:** 2026-08-21

**Authors:** Yiming An, Yanshu Yu, Weidong Li, Orest Kochan

**Affiliations:** 1 Detroit Green Technology Institute, Hubei University of Technology, Wuhan 430068, China; 2 School of Computer Science and Artificial Intelligence, Hubei University of Technology, Wuhan 430068, China; 3 Department of Information-Measuring Technologies, Lviv Polytechnic National University, 79013 Lviv, Ukraine

**Keywords:** binary code similarity detection, candidate reliability auditing, cross-compilation evidence, probability calibration, selective prediction, risk coverage

## Abstract

Binary code similarity detection (BCSD) ranks candidates but does not quantify the reliability of an already selected Top-1 match. We study this post-retrieval problem in a known-source, closed-set protocol: the target Top-1 is frozen before same-source cross-compilation views are queried, so auxiliary evidence audits cannot replace it. A frozen 34-variable map feeds a low-capacity logistic model with project-grouped cross-fitting, Platt calibration, and training-side threshold selection. On 413 families from 16 projects, cross-view evidence improved discrimination over target score/margin features. GCC-O0 was a dominant-anchor regime: Full showed no statistically resolved ROC-AUC gain over Primary-anchor, whereas Clang-O0 benefited from complementary non-primary evidence. On 240 project-identity-disjoint families from 55 projects, the design-locked structural branch accepted 75/240 GCC and 99/240 Clang candidates (31.3%/41.3% coverage) with no observed family-level errors. Correspondence mismatch reduced discrimination toward chance. Corrected TF-IDF remained supportive because correction followed label access. The contribution of this paper is a versioned candidate-preserving audit interface with explicit evidence and deployment boundaries, but not a universal retrieval improvement or distribution-free guarantee.

## 1. Introduction

BCSD supports vulnerability search, patch analysis, reuse analysis, and component identification, yet evaluation commonly ends at pair discrimination or gallery ranking [1,2]. In this study we focus on the study of how reliable a candidate is after a specified query, gallery, and backend produce it. The target search freezes Top-1. Later the compiler views only audit that decision.

For family i, the full-state protocol-conditioned probability of correctness is

(1) $$p_{i}^{*}={Pr}_{\Pi }(Y_{i}=1|Z_{i})$$

where Y_i_ is exact-family correctness, Z_i_ the observable post-freeze state, and Π is the fixed gallery, backend, view roles, feature semantics, estimation scheme, and calibration. This probability is not a raw similarity score: its scale and correctness frequency depend on the protocol, including tie handling and representation collisions.

We evaluate known-source, source-to-binary retrieval: an analyst has a reference source function and can generate controlled query builds whereas its location in a fixed binary gallery is unknown [3]. Unknown-source identification, open-set galleries, cross-architecture transfer, obfuscation, compiler-version shift, and unvalidated missing views are outside of the scope of this protocol.

Our contributions are as follows: (1) protocol-conditioned exact-family correctness for an immutable Top-1 with a versioned candidate-invariance fingerprint; (2) a low-capacity 34-variable auditor separating discrimination, calibration, and selective operating-point validity under project-isolated estimation; and (3) an E27–E39 evidence chain testing anchor sufficiency, collisions/false consensus, design-locked transfer, correspondence disruption, and Direct–Guard–auditor boundaries. The novelty is the audited endpoint and evidence governance, but not the established logistic, Platt, abstention, or Wilson components.

## 2. Related Work

### 2.1. Binary Function Similarity

BCSD includes symbolic/metadata methods [4,5,6,7], graph/sequence encoders [8,9,10,11,12,13], and pretrained or contrastive assembly models [14,15,16,17,18,19,20,21,22,23,24,25]. These optimize pair labels, scores, or rankings and remain sensitive to compiler matrices, leakage, sampling, and gallery construction [18,23,24,25]. Patch verification [3] and UniASM [26] address retrieval performance but not calibrated reliability for an immutable candidate. The transfer of our audit to neural backends remains untested.

### 2.2. Calibration and Selective Prediction

Calibration maps score event probabilities. Platt [27], temperature [28], Dirichlet [29], and isotonic methods have different bias–variance trade-offs. Brier and NLL assess overall probabilistic performance [30]. We use project-isolated Platt calibration because only 16 project clusters are available. Calibrator selection requires another isolated layer. Alternatives were not experimentally compared, so no superiority is claimed.

Selective prediction trades coverage against accepted-set error [31,32,33]. Conformal risk control and learn-then-test provide finite-sample statements under assumptions [34,35], with related work on shift/ranked retrieval [36,37]. Our weaker rule screens training-side probability groups with a one-sided Wilson upper bound and freezes the threshold before held-out-project evaluation. Conformal/LTT comparators were not implemented. Wilson is a family-level diagnostic, not a dependence-robust, project-level, conformal, or distribution-free guarantee.

### 2.3. Reliability Auditing and Minimum-Sufficiency Baselines

Minimum-sufficiency comparisons include target scores, Primary-anchor, Multi-aux-no-primary, support/uniqueness Guards, Direct replacement, and Full. Direct applies when replacement is allowed, Guard provides a transparent fixed-candidate point, and learned auditing adds value for continuous prioritization, multiple thresholds, review costs, or complementary evidence.

### 2.4. Methodological Positioning and Scope

Table 1 positions this study by endpoint, replacement, calibration, abstention, project isolation, compiler/open-set evidence, and guarantee. The contribution of this paper is not a new classifier or calibrator, but a candidate-preserving probability endpoint coupled to versioned cross-compilation evidence and project-isolated decision governance.

## 3. Problem Formulation

### 3.1. Fixed-Candidate Correctness and Protocol-Conditioned Probability

For family i, the frozen target-generated candidate ${\hat{g}}_{i}$ is

(2) $${\hat{g}}_{i}=\rho_{\Lambda }\left(arg\underset{g\in G}{max}s_{M}\left(q_{i}^{0},g\right)\right)$$

where G is the frozen gallery, M is the retrieval backend with the score s_M_, q_i_^0^ is the designated target view, and ρΛ is the deterministic tie rule. The exact-family correctness is

(3) $$Y_{i}=1\{fam\left({\hat{g}}_{i}\right)=fam\left(q_{i}^{0}\right)\}$$

where fam(·) maps a gallery item to its source-function family. Both ${\hat{g}}_{i}$ and Y_i_ are fixed before the auxiliary evidence is encoded. The exact identity is a provenance endpoint, not semantic equivalence.

The versioned prediction protocol is as follows:

(4) $$\Pi_{pred}=\left(G,M,V,\Lambda ,S,C\right)$$

where *V*, *Λ*, 𝒮, and 𝒞 specify view roles, feature/tie semantics, project-isolated estimation, and calibration, respectively; *Π_de_*_c_ extends *Π_pred_* by additionally fixing the candidate threshold set, the nominal risk budget α, and the upper-tail probability δ. The protocol-conditioned reliability is

(5) $$\eta_{\Pi }\left(x\right)=\underset{\Pi }{Pr}\left(Y=1|X=x\right).$$

Because X = ΦΛ(Z) compresses the observable state, ηΠ(X) = EΠ[p*|X]. Compression can discard but not create conditional information. The states with different correctness that map to the same X cannot be separated by an X-measurable predictor. Byte collisions and stable false consensus are the therefore identifiability limits. Table 2 fixes notation.

### 3.2. Task Hierarchy and Candidate Invariance

Pairwise detection labels a pair, retrieval establishes a ranking on G, Direct may replace the target result, and auditing estimates the correctness of the already frozen target candidate. Therefore, candidate invariance fixes the statistical event before auxiliary views are inspected.

(6) $$U_{i}=\left({\hat{g}}_{i},{\hat{p}}_{i},A_{\tau ,i}\right),\;\;A_{\tau ,i}=1\{{\hat{p}}_{i}\geq \tau \}$$

At threshold τ, the online output U_i_ returns (${\hat{g}}_{i}$, ${\hat{p}}_{i}$) with an accept/abstain indicator, Aτ,_i_; abstention suppresses an automatic action but preserves the candidate and probability for review.

### 3.3. Hierarchical Estimands and Non-Interchangeable Risks

Families are observational units and projects are dependence clusters. We therefore report family-pooled accepted risk, equal-project macro risk, and maximum observed project risk as defined in Equations (7) and (8).

(7) $$R_{fam}\left(\tau \right)=\frac{\sum_{p}e_{p}\left(\tau \right)}{\sum_{p}n_{p}\left(\tau \right)},\;\;R_{macro}\left(\tau \right)=\frac{1}{\left|P_{acc}\right|}\sum_{p\in P_{acc}}\frac{e_{p}\left(\tau \right)}{n_{p}\left(\tau \right)},$$

(8) $$R_{max}\left(\tau \right)=\underset{p\in P_{acc}}{max}\frac{e_{p}\left(\tau \right)}{n_{p}\left(\tau \right)}.$$

Here, n_p_(τ) and e_p_(τ) are accepted and erroneous counts in project p, respectively; Pacc(τ) contains projects with nonzero acceptance. Rfam weights families, Rmacro weights projects equally, and Rmax is a heterogeneity diagnostic, not a population worst-case guarantee.

### 3.4. Identifiability and Falsifiable Claims

Monotone score transforms preserve rank but alters the probability scale, while opposite labels can share identical evidence. Ties, byte-equivalence groups, and stable false consensus therefore mark information limits, not merely insufficient model capacity.

(9) $$Pr\left(Y=1|X_{0},W\right)\neq Pr\left(Y=1|X_{0}\right)$$

For the existing evidence X_0_ and added block W, Equation (9) requires W to change conditional correctness on a positive-probability set. Anchor ablation, support strata, collision/challenge subsets, and correspondence mismatch operationalize this criterion.

Proposition (selective ordering): For a fixed protocol and feasible coverage c, accepting an upper level set of ηΠ(X) minimizes expected accepted-set error among X-measurable rules. Swapping an accepted lower-η state for a rejected higher-η state cannot increase error. ROC-AUC/AURC therefore assess ordering, whereas Brier/NLL assess probabilistic performance; neither alone validates a transferred risk threshold.

## 4. Candidate-Preserving Reliability Auditor

### 4.1. Architecture, Observable State, and Invariants

**Figure 1:**
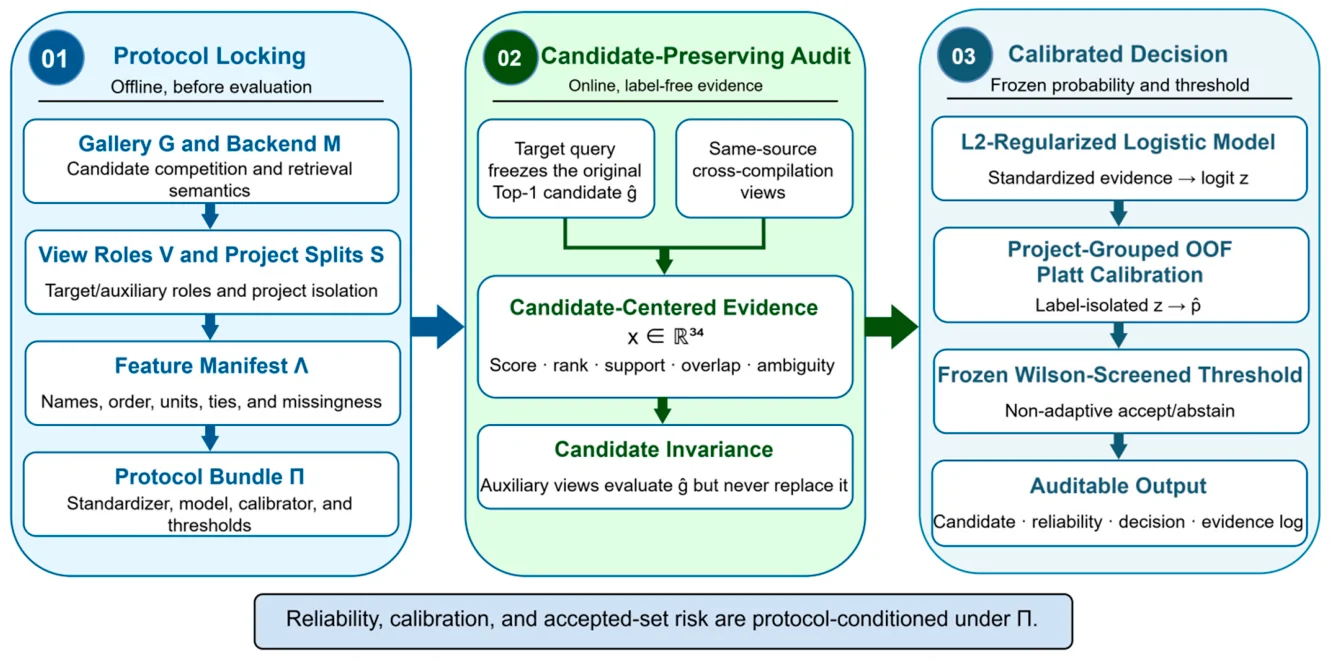
Candidate-preserving audit. Stage 01 locks the protocol bundle; Stage 02 freezes the target Top-1 and evaluates that candidate across auxiliary views without replacement; Stage 03 returns calibrated reliability with an accept/abstain decision.

At deployment, the offline bundle is immutable. Protocol mismatch triggers abstention rather than reuse of an invalid probability map. Figure 1 shows the candidate-preserving audit workflow.

The candidate-centered encoder is as follows:

(10) $$X_{i}=\Phi_{\Lambda }\left(Z_{i}\right)$$

where Z_i_ contains the frozen candidate, binary views, retrieval traces, ties, byte-equivalence groups, and missingness; ΦΛ cannot access Y_i_, family identity, or outcome-derived fields.

Computational identity is protected by the SHA-256 protocol fingerprint:

(11) $$F\left(\Pi \right)=SHA256\{canon\left(G,M,V,\Lambda ,S,\beta ,a,b,\tau \right)\}$$

where canon(·) deterministically serializes the frozen components, coefficients β, the Platt parameters (a, b), and the threshold τ. Fingerprint equality establishes implementation identity, but not distributional validity.

### 4.2. Frozen 34-Dimensional Candidate-Centered Evidence

**Figure 2:**
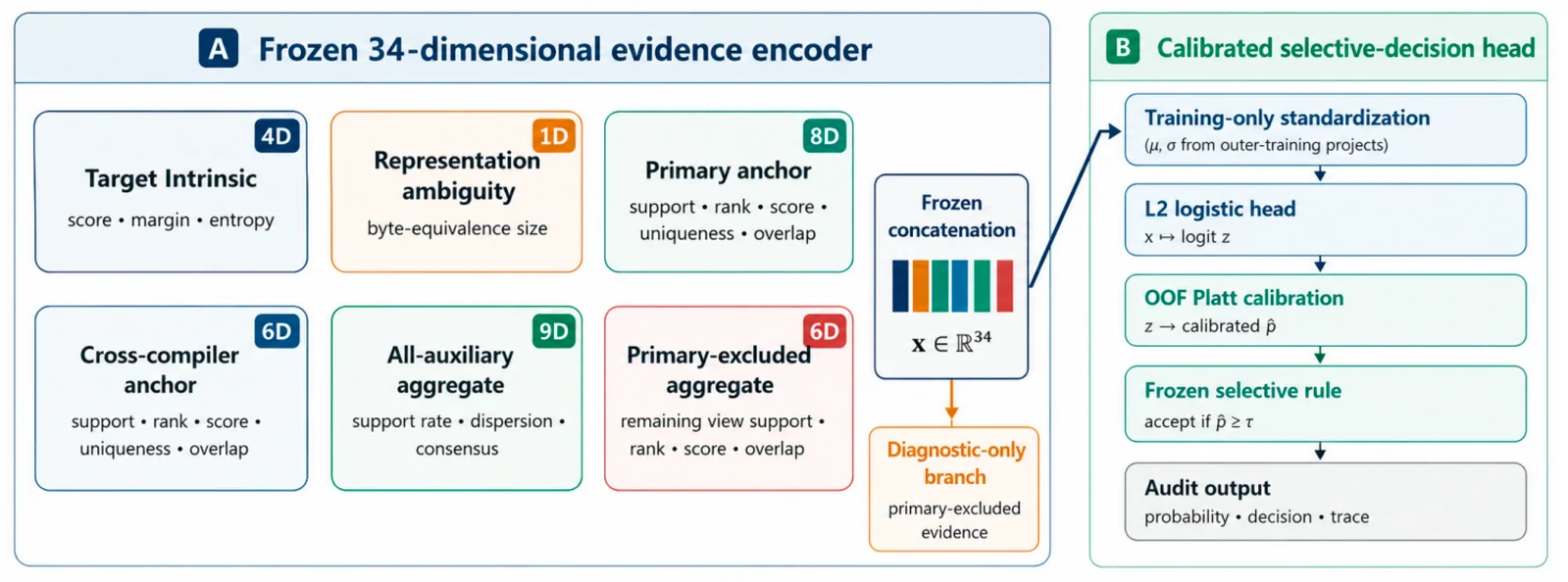
The frozen 34-dimensional evidence schema and calibrated decision head. The no-primary aggregate supports anchor-dependence diagnostics.

Figure 2 shows the frozen 34-dimensional evidence schema and calibrated decision head. Equation (12) concatenates the six evidence blocks. Equation (13) defines normalized target-score weights and entropy.

(12) $$X_{i}=\left[X_{i}^{tar},X_{i}^{amb},X_{i}^{pri},X_{i}^{cross},X_{i}^{all},X_{i}^{no\text{-}pri}\right]\in R^{34}$$

Superscripts tar, amb, pri, cross, all, and no-pri denote the target, ambiguity, Primary-anchor, cross-compiler, all-auxiliary, and primary-excluded blocks, respectively.

(13) $$w_{ij}=\frac{exp\left(s_{ij}/T\right)}{\sum_{l=1}^{L}exp\left(s_{il}/T\right)},\;\;H_{i}=-\frac{1}{logL}\sum_{j=1}^{L}w_{ij}logw_{ij}$$

For retained target scores s_ij_, T is the frozen temperature, L is the retained count, w_ij_ is the normalized weight, and H_i_ is entropy normalized by log L. We use the values of T = 1 and L = 20 in this article.

Auxiliary evidence comprises candidate rank (14), Top-1 support/reciprocal rank (15), target–auxiliary Top-k Jaccard overlap (16), and cross-view rank/support/uniqueness aggregates (17)–(18).

(14) $$r_{iv}=1+\sum_{\begin{array}{c} g\in G\\ g\neq {\hat{g}}_{i} \end{array}}I\;\left(g{≻}_{v}{\hat{g}}_{i}\right)$$

(15) $$a_{iv}=1\{r_{iv}=1\},\;\;rr_{iv}=r_{iv}^{-1}$$

(16) $$J_{iv}^{\left(k\right)}=\frac{\left|N_{i}^{0}\left(k\right)\cap N_{i}^{v}\left(k\right)\right|}{\left|N_{i}^{0}\left(k\right)\cup N_{i}^{v}\left(k\right)\right|},\;\;k\in \{5,10\}$$

(17) $${\bar{a}}_{i}=\frac{1}{\left|\nu_{i}\right|}\sum_{v\in \nu_{i}}a_{iv},\;r_{i}^{min}=\underset{v}{min}r_{iv},\;{\bar{r}}_{i}=\frac{1}{\left|\nu_{i}\right|}\sum_{v}r_{iv}$$

(18) $${\bar{u}}_{i}=\frac{1}{\left|\nu_{i}\right|}\sum_{v}u_{iv},\;s_{u,i}={\left\{\frac{1}{\left|\nu_{i}\right|-1}\sum_{v}{\left(u_{iv}-{\bar{u}}_{i}\right)}^{2}\right\}}^{1/2},\;{\bar{J}}_{i}^{\left(k\right)}=\frac{1}{\left|\nu_{i}\right|}\sum_{v}J_{iv}^{\left(k\right)}$$

Here, a_iv_ is support, r_iv_/rr_iv_ is rank/reciprocal rank, u_iv_ is uniqueness, and J_iv_(k) is Top-k ID-set overlap for k ∈ {5, 10}. Table 3 allocates the 34 variables. Correlated fields are not interpreted causally. Ablation, collision, challenge, and mismatch analyses test the mechanism.

### 4.3. Low-Capacity Probability Model

The low-capacity base model minimizes the standardized ℓ_2_-regularized logistic objective in Equation (19).

(19) $$\left({\hat{\beta }}_{0},\hat{\beta }\right)=arg\underset{\beta_{0},\beta }{min}\left[\sum_{i\in I_{tr}}log\{1+exp\left[-t_{i}\left(\beta_{0}+\beta^{⊤}{\tilde{x}}_{i}\right)\right]\}+\frac{\lambda }{2}{∥\beta ∥}_{2}^{2}\right]$$

The frozen implementation uses z-score standardization, C = 1, balanced class weights, L-BFGS, and the tolerance of 10^−4^. Every scaler and fitted parameter is estimated only on the corresponding training partition.

### 4.4. Nested Project Cross-Fitting and Platt Calibration

Project isolation is nested. The outer-test projects are not subjected to standardization, fitting, calibration, and threshold selection. The inner-project OOF logits fit the Platt map in Equation (20). The base model is then refitted on all outer-training projects and Equation (21) calibrates held-out logits.

(20) $$\left({\hat{a}}^{\left(-k\right)},{\hat{b}}^{\left(-k\right)}\right)=arg\underset{a,b}{min}-\sum_{j\in I_{-k}}\left[Y_{j}log\sigma \left(az_{j}^{in}+b\right)+\left(1-Y_{j}\right)log\{1-\sigma \left(az_{j}^{in}+b\right)\}\right]$$

Parameters a_−k_ and b_−k_ use inner-OOF logits only. Equations (22)–(26) define Brier/NLL, ECE-10, project calibration residual/MACR, tie-aware AURC, and the nested operator.

(21) $${\hat{p}}_{i}=\sigma \left({\hat{a}}^{\left(-k\right)}z_{i}^{\left(-k\right)}+{\hat{b}}^{\left(-k\right)}\right),\;\;i\in P_{k}$$

(22) $$Brier=\frac{1}{n}\sum_{i=1}^{n}{\left({\hat{p}}_{i}-Y_{i}\right)}^{2},\;\;NLL=-\frac{1}{n}\sum_{i=1}^{n}\left[Y_{i}log{\hat{p}}_{i}+\left(1-Y_{i}\right)log\left(1-{\hat{p}}_{i}\right)\right]$$

(23) $$ECE=\sum_{b}\frac{\left|B_{b}\right|}{n}\left|\frac{1}{\left|B_{b}\right|}\sum_{i\in B_{b}}{\hat{p}}_{i}-\frac{1}{\left|B_{b}\right|}\sum_{i\in B_{b}}Y_{i}\right|$$

(24) $$g_{p}=\frac{1}{n_{p}}\sum_{i\in p}\left({\hat{p}}_{i}-Y_{i}\right),\;\;MACR=\frac{1}{\left|P\right|}\sum_{p\in P}\left|g_{p}\right|$$

(25) $$AURC=\sum_{j=1}^{J}\frac{m_{j}-m_{j-1}}{n}\frac{E_{j}}{m_{j}},\;\;m_{0}=0$$

(26) $${\hat{p}}_{i}^{\left(k\right)}=\left(C_{-k}\circ A_{-k}\right)\left(X_{i}\right),\;\;i\in P_{k}$$

No outer-project label enters fitted models, calibrators, or thresholds. The outer-project observations do not estimate standardization or model parameters. Equal probabilities remain grouped. Inner-OOF calibration, outer evaluation, and all-history refitting are distinct; the nested design limits information leakage but does not eliminate uncertainty associated with having only 16 projects.

### 4.5. Selective Acceptance, Finite-Sample Bounds, and Threshold Freezing

Equation (27) defines acceptance, coverage, and accepted risk for frozen threshold τ. Equation (28) is for the one-sided Wilson upper bound [39]. Equation (29) is responsible for training-side threshold selection.

(27) $$A_{\tau ,i}=1\{{\hat{p}}_{i}\geq \tau \},\;\;C_{\tau }=\frac{1}{n}\sum_{i=1}^{n}A_{\tau ,i},\;\;R_{\tau }=\frac{\sum_{i=1}^{n}A_{\tau ,i}\left(1-Y_{i}\right)}{\sum_{i=1}^{n}A_{\tau ,i}}$$

(28) $$U_{W}\left(n,e;\delta \right)=\frac{\hat{r}+\frac{z_{1-\delta }^{2}}{2n}+z_{1-\delta }\sqrt{\frac{\hat{r}\left(1-\hat{r}\right)}{n}+\frac{z_{1-\delta }^{2}}{4n^{2}}}}{1+\frac{z_{1-\delta }^{2}}{n}},\;\;\hat{r}=\frac{e}{n}$$

(29) $$\hat{\tau }=arg\underset{\tau \in T}{max}C_{in}\left(\tau \right)\;s.t.\;U_{W}\;\left(n_{in}\left(\tau \right),e_{in}\left(\tau \right);\delta \right)\leq \alpha$$

For n accepted cases and e errors, z_1−δ_ is the one-sided standard-normal quantile. Equation (29) maximizes attainable inner-OOF coverage under working budget α; equal-probability groups remain joint and reject-all is always feasible.

Wilson uses the family-level Bernoulli model and is not robust to within-project dependence or a project-level/distribution-free guarantee. Therefore, we report repeated project partitions, cluster resampling, Rmacro and Rmax alongside counts, coverage, and empirical risk. Zero observed errors do not imply zero deployment risk.

Equation (30) decomposes accepted risk into probability-implied risk and selective calibration residual. A numerical probability threshold is therefore not itself a risk bound.

(30) $$R_{\tau }=E\left[1-Y|A_{\tau }=1\right]=E\left[1-\hat{p}|A_{\tau }=1\right]+E\left[\hat{p}-Y|A_{\tau }=1\right]$$

### 4.6. Decision Utility, Protocol Matching, and Computational Scope

Evidence acquisition is costly: target-only retrieval uses one gallery search, Primary-support/Guard two, and locked Full five, plus auxiliary compilation/disassembly. We claim no end-to-end latency, storage, energy, or cost advantage. Direct GCC-O3 was simpler and more accurate when replacement was allowed.

Protocol shift can invalidate the probability map. For an acceptance event A with probability at least c_min_ under Π and Π′, Equation (31) bounds the accepted-risk change by total-variation distance.

(31) $$\left|R_{\Pi^{\prime }}\left(A\right)-R_{\Pi }\left(A\right)\right|\leq \frac{2d_{TV}\left(P_{\Pi^{\prime }},P_{\Pi }\right)}{c_{min}}$$

Because dTV(PΠ′,PΠ) is not estimated, Equation (31) motivates re-certification rather than certifying deployment.

If false acceptance cost is C_e_ and manual review cost is C_r_, then the Bayes acceptance condition is given by Equation (32), as follows:

(32) $$\left(1-\hat{p}\right)C_{e}\leq C_{r}\;\Leftrightarrow \;\hat{p}\geq 1-\frac{C_{r}}{C_{e}}.$$

For 0 < Cr < Ce, the probability threshold is 1 − Cr/Ce. Thus, one calibrated score can support application-specific review policies even when a transparent Guard is competitive at one point. We make no cost-effectiveness claim.

Cyclic mismatch preserves candidates, labels, projects, fitted artifacts, thresholds, and feature marginals while breaking candidate–auxiliary correspondence. Loss of discrimination is therefore a falsificatory alignment control, but not a causal intervention.

Within the evaluated protocol, Direct is simplest when replacement is allowed. Guard suits one transparent fixed-candidate point. Learned auditing suits calibrated prioritization, multiple thresholds, review costs, or complementary evidence. Table 4 and Table 5 summarize the procedures.

## 5. Experimental Design

### 5.1. Data Construction and Evidence Levels

We built a six-view x86-64 matrix from AnghaBench [38] using GCC 15.1.0 and Clang 22.1.6 at O0/O2/O3, disassembled with GNU objdump 2.44. A 91-family mechanism cohort examined candidate freezing, anchor dominance, false consensus, uniqueness, and collisions. It is a controlled benchmark, but not a population-representative software sample.

The experiment ledger retains E1–E39. E1–E26 were exploratory protocol-development runs and are not used for confirmatory inference. E27–E39 are organized by evidential role, with chronology in Supplementary Data S1.

Preflight screened 10,074 files. Out of 516 attempted candidates, 500 families from 19 projects passed all six builds and representation QA (3000 views). Outcome-independent reduction to 413 families removed 20 development overlaps, 54 short-only cases, 9 representation/symbol-only cases, 2 meeting both criteria, and 2 prespecified high-risk paths. The 81-family challenge manifests overlaps with the primary cohort in 33 families and does not represent all 87 exclusions.

Table 6 and Table 7 summarize cohort construction and evidence roles. Eligibility required complete six-view compilation and valid target/gallery representations. Retrieval scores, predictions, labels, and model metrics were excluded from eligibility decisions. Portability changes were confined to the benchmark platform-typedef scaffold, left target function bodies unchanged, and were hash-audited. Projects remained indivisible and build/QA/exclusion decisions were fixed before modeling.

For identity-disjoint transfer, 278 candidates from 56 projects were attempted and 240 QA-qualified families from 55 projects yielded 1440 views. No project name or raw/normalized source hash overlapped with earlier data. Functions spanned 3–403 instructions (median 27), including one six-view byte-identical cross-family pair. This tests unseen project identities within the same ecosystem and toolchain, but not an independent external ecosystem.

### 5.2. Retrieval Protocols and Baselines

Each closed-set gallery used one GCC-O2 representation per family (413 internal; 240 transfer). Targets were GCC-O0 and Clang-O0, with GCC-O3 as the prespecified primary anchor. TF-IDF used normalized assembly, small constants, Top-20 entropy (T = 1), and Top-5/10 overlap. The development-only search over 54 structural configurations locked raw third-order signatures, IDF overlap, Dice similarity, and unit weights. BM25/Jaccard were exploratory stress tests.

Learned comparators were as follows: Score-only, Score + Margin, Primary-anchor, Multi-aux-no-primary, and Full, with identical project folds, training-only standardization, optimization, inner-OOF calibration, and threshold search. Support/Guard preserve the frozen target. Direct GCC-O3 may replace it and therefore uses a different estimand.

### 5.3. Metrics, Uncertainty, and Evidence Governance

Primary metrics were as follows: ROC-AUC, Brier, NLL, and tie-aware AURC for exact-family correctness. PR-AUC and ECE-10 were supplementary. Critical contrasts were Full versus Score + Margin and versus the strongest minimum-sufficiency branch. Paired 1000-replicate project-cluster bootstrap intervals [40] resampled projects as clusters. With only 16 project clusters, these intervals are cluster-sensitive uncertainty summaries, not high-precision asymptotic statements.

Nested evaluation used five outer project folds, ten repetitions, and three inner folds. Each frozen operating point reports accepted/error counts, coverage, empirical risk, one-sided Wilson diagnostic and compliance frequency, plus family-pooled, project-macro, and maximum observed project risks.

Supplementary File S1 contains representative trajectories, Tables S1–S4, and Figures S1–S4. Supplementary Data S1 contains the evidence ledger, candidate diagnostics, operating points, bootstrap summaries, split audits, and archive manifest. Figures were regenerated from archived numerical outputs and did not feed back into model, threshold, or cohort selection.

Randomized correspondence controls use the add-one permutation *p*-value given in Equation (33).

(33) $$p_{perm}=\frac{1+\sum_{b=1}^{B}1\{T_{b}\geq T_{obs}\}}{B+1}$$

With B permutations, T_obs_ is the aligned statistic and T_b_ its permuted value; within-project cyclic mismatch is a separate deterministic negative control.

Evidence roles are non-interchangeable: mechanism/qualification, internal validation, held-out operating points, design-locked transfer, supportive correction, and post hoc boundary analysis. Here, “locked” denotes time-stamped choices fixed before the corresponding outcome evaluation; it is not equivalent to prospective preregistration.

### 5.4. Supportive Heterogeneity and Decision Analyses

Because 413 families arise from 16 projects, project composition is a major uncertainty source. For frozen OOF predictions, Equation (34) defines leave-one-project-out composition influence.

(34) $$I_{p,m}=\theta_{m}\left(D_{-p}\right)-\theta_{m}\left(D\right),\;\;D_{-p}=D\backslash D_{p}$$

Here, D is the frozen OOF set, D−p removes project p, and θm is metric m. Models are not refitted, so Ip,m measures summary sensitivity to project composition. The corresponding absolute changes are reported in Section 6.7 below.

Project calibration is summarized by MACR and upper project residuals. Equation (35) shows the coexistence of a small pooled signed residual with larger project-wise errors.

(35) $$g_{pool}=\sum_{p\in P}\pi_{p}g_{p},\;\;\left|g_{pool}\right|\leq \sum_{p\in P}\pi_{p}\left|g_{p}\right|,\;\;MACR=\frac{1}{\left|P\right|}\sum_{p\in P}\left|g_{p}\right|$$

Here, g_p_ is project p’s signed calibration residual and π_p_ its family weight. Project-specific calibration slopes are not fitted because several projects are small or single-class.

## 6. Results

### 6.1. Mechanism Studies and Retrieval Difficulty

In the study of the 91-family mechanism, target R@1 was 0.1978/0.0769 for GCC/Clang versus 0.9451 for GCC-O3. Within this development set, GCC-O3 support exactly matched correctness for both target tasks. Its removal reduced GCC discrimination much more than Clang discrimination. This is consistent with dominant-anchor behavior but does not establish a general multi-view advantage.

Two stable false-consensus cases remained. The uniqueness Guard removed observed development errors at lower coverage. Five cross-source byte-collision pairs produced 30 records across six compiler configurations, with raw signatures differing in only 9/30. These findings motivate ambiguity/uniqueness evidence without redefining exact-family correctness.

The 413-family primary cohort was harder (Table 8): target R@1 was 0.5061/0.4310, while GCC-O3 reached 0.9709, motivating the dominant-anchor analysis.

### 6.2. Primary Project-Isolated Reliability Performance

**Figure 3:**
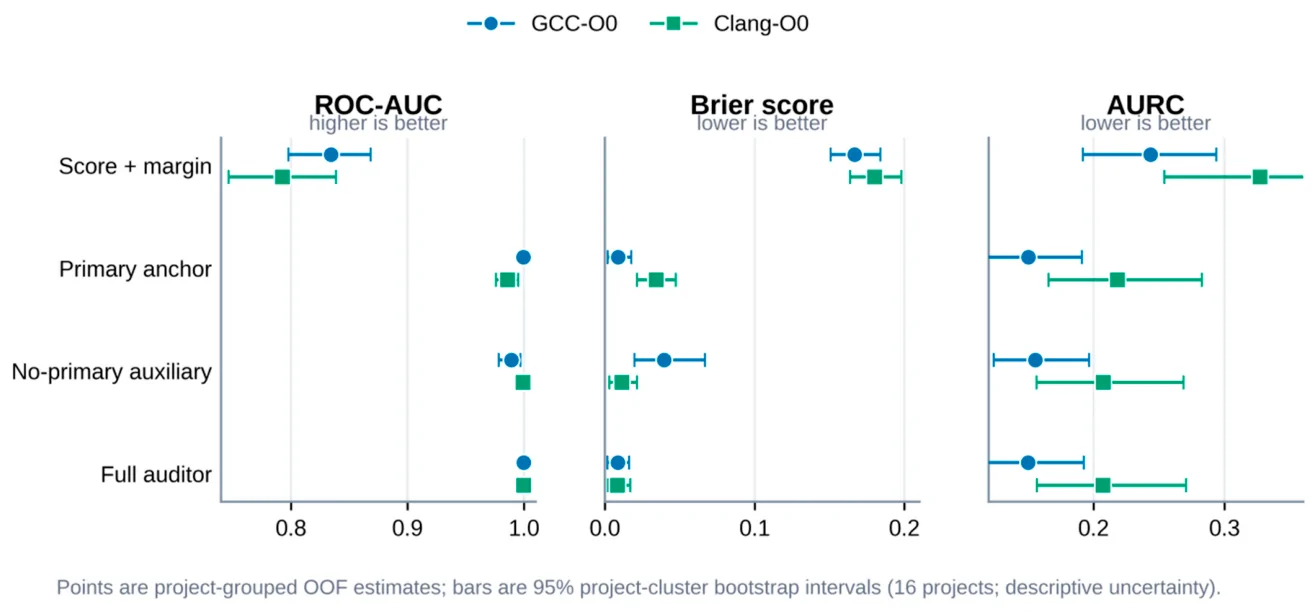
Project-grouped OOF performance on 413 families (16 projects), with 95% project-cluster bootstrap intervals for ROC-AUC, Brier, and AURC.

Both Figure 3 and Table 9 show that cross-view models improved discrimination over Score + Margin but Full was not uniformly necessary. For GCC, Full’s ROC-AUC gain over Primary-anchor was not statistically significant. For Clang, Full improved over Primary-anchor but did not improve significantly over Multi-aux-no-primary. Complementarity is therefore task-specific, not evidence of universal 34-variable superiority.

At diagnostic points, Primary-support accepted 218/413 GCC and 169/413 Clang candidates, with nine errors each. Uniqueness Guard accepted 197/413 GCC with zero errors and 153/413 Clang with two. Retrospectively matching Full to Guard coverage also gave zero errors in both tasks. These remain internal OOF diagnostics, not frozen deployment points.

In frozen OOF predictions, Full also reduced observed residuals of project calibration. With respect to Score + Margin, MACR fell from 0.0769 to 0.0031 (GCC) and 0.1187 to 0.0089 (Clang). The maximum Full project residuals were 0.0154/0.0452. Intervals and project counts are in Supplementary Data S1.

### 6.3. Incremental Evidence and Strong-Anchor Asymmetry

Table 10 shows the asymmetry. For GCC, primary support covered every correct candidate plus nine incorrect ones. Full versus Primary-anchor gave ΔROC-AUC = 0.0002 (95% CI −0.0002–0.0007), which is not statistically significant. For Clang, 18 correct candidates lacked primary support. Full versus Primary-anchor improved by 0.0139 (0.0051–0.0241), while the gain over Multi-aux-no-primary was not statistically significant.

$$\eta \left(A,W\right)=A\;\eta_{1}\left(W\right)+\left(1-A\right)\;\eta_{0}\left(W\right),\;\;\eta_{a}\left(W\right)=Pr\left(Y=1|A=a,W\right)$$

Let A denote primary support and W all remaining evidence. No correct GCC candidate occurred at A = 0, so W mainly resolves false consensus within A = 1. In Clang, 18 correct candidates occurred at A = 0, allowing non-primary evidence to recover candidates that primary support would reject.

All 195 unsupported GCC candidates were incorrect, whereas 18/244 unsupported Clang candidates were correct. Multi-aux-no-primary removes GCC-O3 and is refitted/recalibrated on remaining prespecified views. It tests anchor dependence, but not arbitrary runtime missingness, which requires a separately validated subprotocol.

### 6.4. Nested Frozen Operating Points

**Figure 4:**
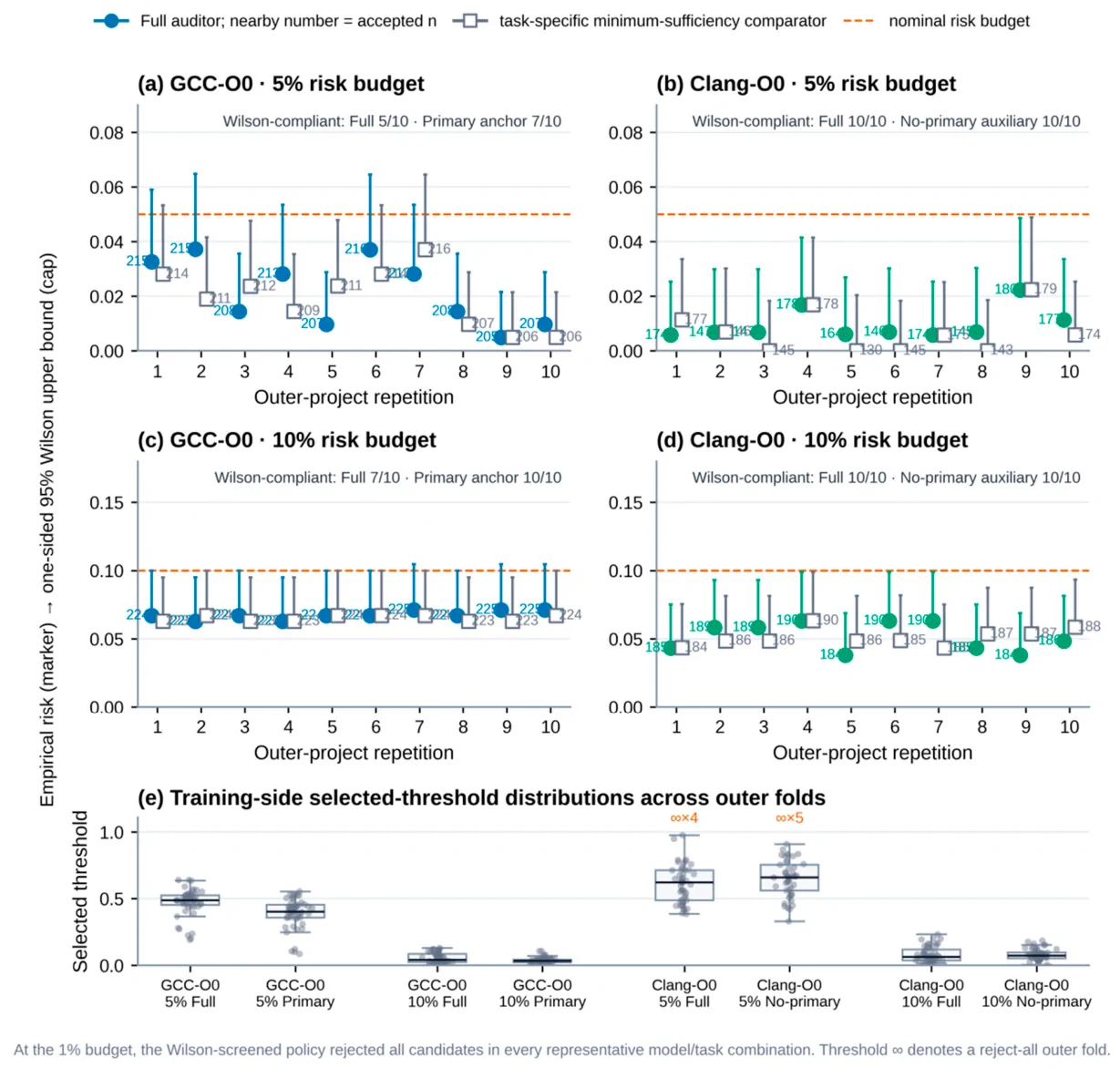
The diagnostics of nested operating-point: Outer-project accepted risk, 95% Wilson upper bounds, accepted counts, and training-side threshold distributions. The sign ∞ denotes reject-all.

Figure 4 and Table 11 show that all 1% policies selected reject-all. At 5%, the held-out Wilson diagnostic was met in 10/10 repetitions for Clang Full, 5/10 for GCC Full, and 7/10 for GCC Primary-anchor. Thus, strong discrimination did not ensure stable threshold transfer. Repetition-level thresholds, counts, risks, bounds, Rfam, Rmacro, and Rmax are in Supplementary Data S1.

### 6.5. Cross-Backend and Identity-Disjoint Transfer

The design-locked structural branch provides outcome-untouched transfer evidence (Table 12). On 240 project-identity-disjoint families, target for R@1 was 0.3125/0.4333 (GCC/Clang). The selected models had observed ROC-AUC = 1.0000, with frozen acceptance of 75/240 and 99/240, respectively. Original locked TF-IDF failed (182/135 and 240/142 accepted/errors). After label access, correcting target entropy yielded 47/0 and 98/0, respectively. These results are supportive, not confirmatory.

Structural Wilson upper bounds were 0.0348/0.0266. The corrected TF-IDF gave 0.0544/0.0269, so GCC missed the nominal 5% screen. Identity-disjoint ROC-AUC = 1.0000 was largely explained by one support variable, indicating protocol-specific alignment rather than a universal transfer. The cohort still shares AnghaBench, x86-64, compiler matrix, complete-view QA, and pipeline assumptions with development data.

The structural configuration was fixed before outcome evaluation on the 240-family cohort. TF-IDF correction followed label access. Candidates, labels, other features, models, calibrators, and thresholds were unchanged, yet the corrected branch remains supportive only.

### 6.6. Correspondence Mechanism and System Boundary

**Figure 5:**
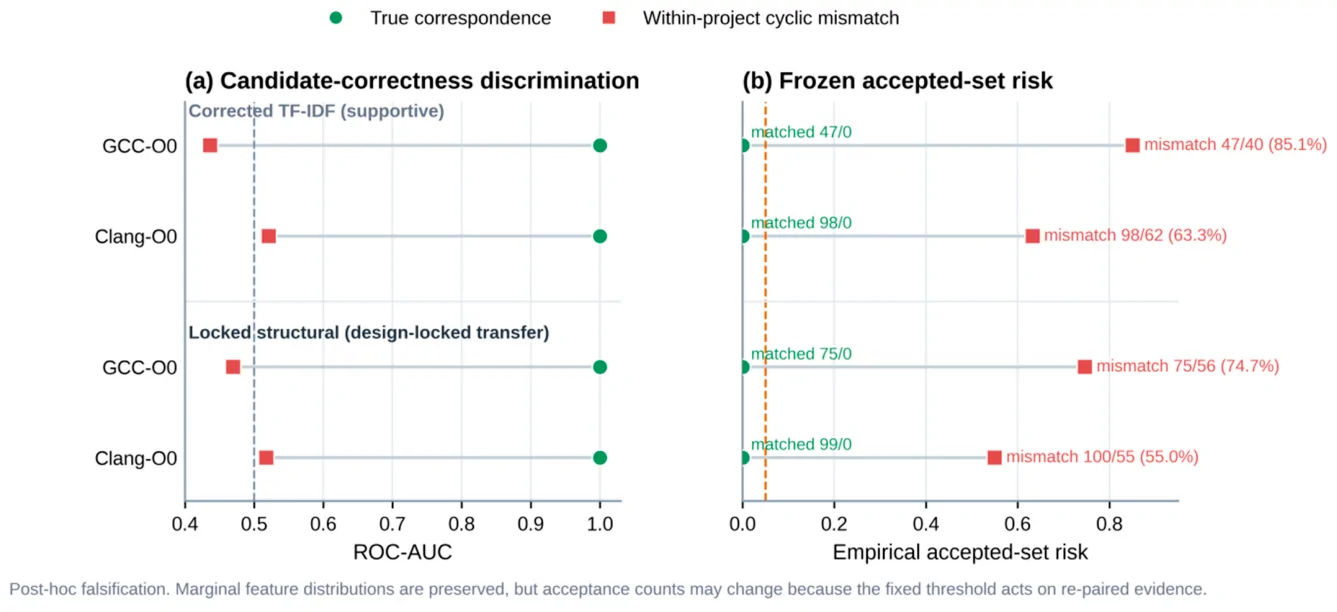
Post hoc correspondence falsification on 240 identity-disjoint families: Aligned versus within-project cyclic mismatch. Structural results are design-locked and corrected TF-IDF supportive.

Figure 5 and Table 13 show add-one permutation *p* = 1/1001. Within-project mismatch reduced ROC-AUC toward chance (0.436–0.521 TF-IDF; 0.470–0.517 structural) and raised accepted risk to 0.55–0.85. Structural Clang accepted 99 candidates aligned and 100 after mismatch because the threshold stayed frozen while feature pairing changed.

**Figure 6:**
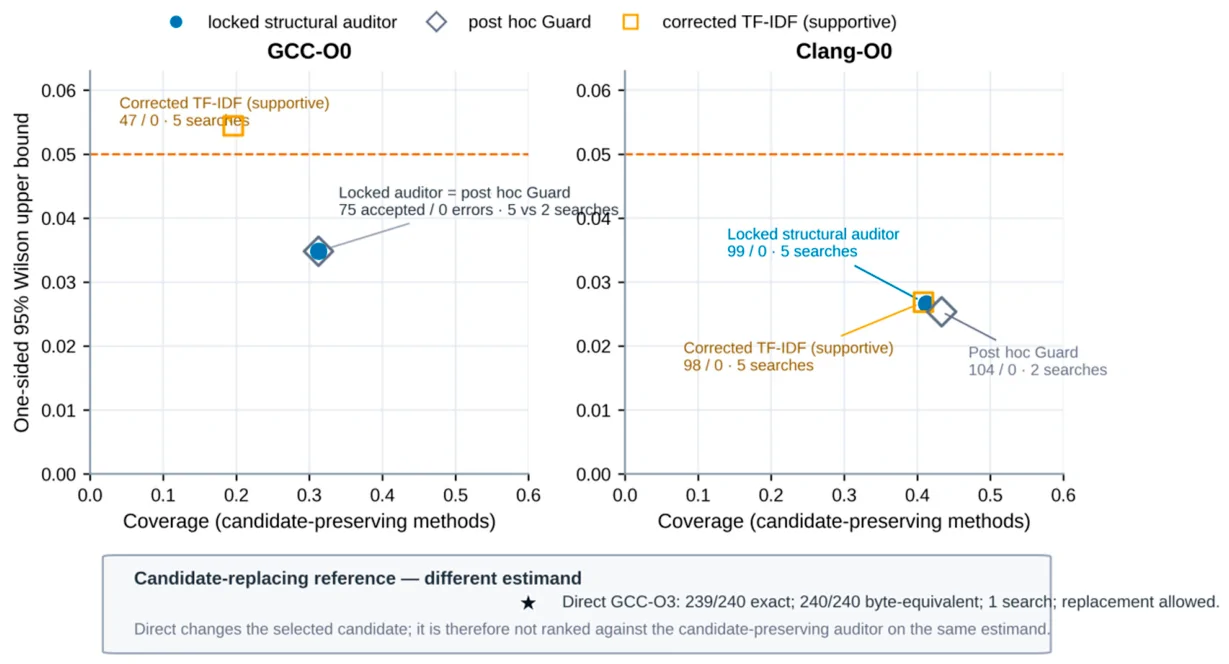
Identity-disjoint system boundaries: Coverage and one-sided 95% Wilson upper bounds for structural auditor, post hoc Guard, supportive corrected TF-IDF, and separate Direct replacement.

Figure 6 shows that Direct GCC-O3 returned 239/240 exact-family and 240/240 byte-equivalent matches when replacement was allowed. Structural Guard matched locked Primary-anchor at 75/240 GCC candidates with zero errors and accepted 104/240 Clang with zero errors versus 99/240 for locked Full. Because Guard was evaluated post hoc on the fresh cohort, it cannot supersede the locked endpoint; it only shows that learned fusion is not uniformly necessary.

The minimum-sufficiency comparison separates three roles: Direct for replacement, a prospectively specified Guard for one transparent operating point, and learned auditing for calibrated prioritization, multiple thresholds, review costs, or complementary evidence.

### 6.7. Candidate Trajectories, Challenge Set, and Local Calibration

Candidate trajectories show stable support, false consensus, byte collision, and cross-view conflict. Supplementary File S1 provides representative cases and Supplementary Data S1 the records. The patterns denote consistent support, persistent support for an incorrect family, cross-family representation ambiguity, and conflicting rank/support evidence, respectively.

**Figure 7:**
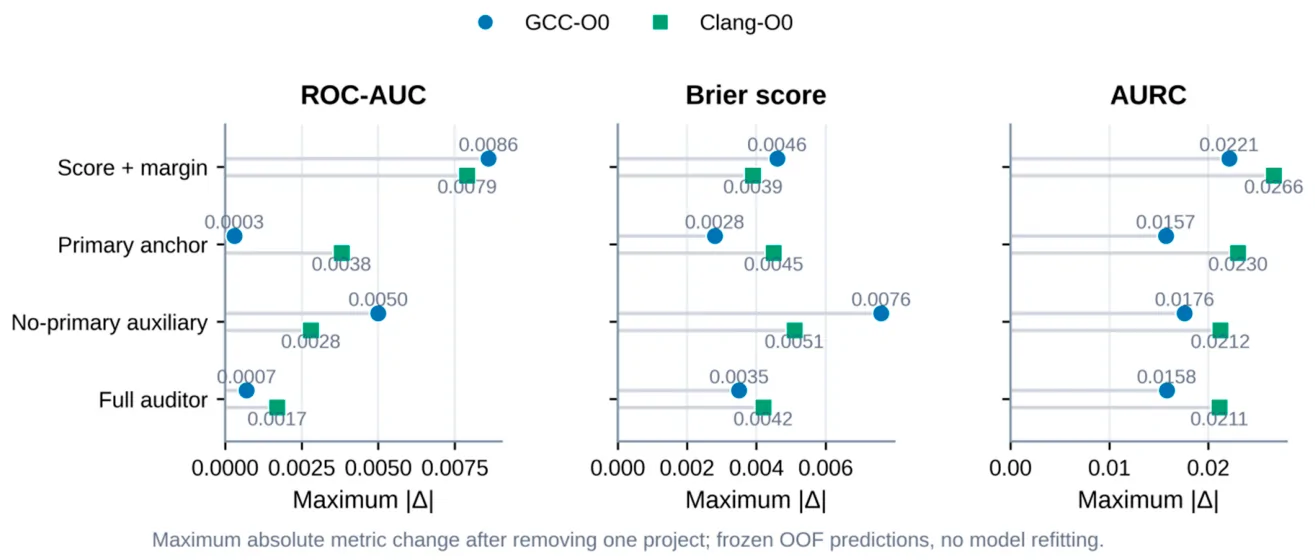
Leave-one-project-out sensitivity of frozen ROC-AUC, Brier, and AURC. The models were not refitted.

On the 33-family challenge intersection, Full ROC-AUC was 0.9325/0.9545, Brier 0.0966/0.0856, and AURC 0.3078/0.3283 (GCC/Clang). Guard had no feasible 1%, 5%, or 10% point. The auditor can prioritize ambiguity but cannot resolve distinctions absent from the evidence. Figure 7 reports project-composition sensitivity.

Cross-task reuse preserved some ordering (ROC-AUC 0.9286/0.9132) but worsened probabilistic performance (Brier 0.1364/0.1424), showing that probability-scale performance does not automatically transfer across target tasks.

## 7. Discussion

Retrieval quality and post-retrieval auditing are distinct. Target–auxiliary correspondence was highly informative but support-dependent: GCC-O3 nearly eliminated unsupported positives, so extra evidence mainly filtered false consensus. Clang retained unsupported true candidates recoverable by other views. Comparable Full ROC-AUC values may therefore arise from distinct evidence mechanisms, underscoring the need for strong simple comparators as minimum-sufficiency baselines.

Discrimination, calibration, and decision validity are non-interchangeable. Near-perfect ordering coexists with project-sensitive thresholds, and Wilson remains a family-level diagnostic. We therefore report coverage, empirical and hierarchical risks, cluster resampling, and project-composition sensitivity together. Zero observed errors are not considered as zero deployment risk.

Project-identity-disjoint transfer removes project-name/source-hash overlap but retains AnghaBench, x86-64, the compiler matrix, controlled builds, complete views, and a closed gallery. The design-locked structural branch is outcome-untouched transfer evidence. Corrected TF-IDF is supportive because target-entropy repair followed label access. No second untouched cohort was available for prospective re-evaluation.

Within this setting, the minimum sufficiency favors Direct when replacement is allowed, a prospectively specified Guard for one transparent fixed-candidate point, and learned auditing when continuous ranking, multiple thresholds, review costs, or complementary evidence are required.

## 8. Threats to Validity

Table 14 summarizes the study. Exact identity does not imply semantic equivalence. The 413 families are clustered within 16 projects, and the challenge subset is not representative of all exclusions. The 240-family cohort is project-identity-disjoint, not ecosystem-independent. No-primary removes one prespecified view rather than testing arbitrary missingness. Open-set retrieval, architecture/compiler shifts, obfuscation, and neural backends require separate validation. Corrected TF-IDF lacks confirmation on a second untouched dataset, and alternative calibration/formal risk-control methods were not compared. We claim neither Platt superiority nor conformal, project-robust, or distribution-free guarantees.

Mechanism studies, corrected TF-IDF, and post hoc Guard/system analyses are not independent confirmations. The study was not prospectively preregistered. The time-stamped design locks document provenance but does not constitute preregistration. Supplementary reporting supports auditability. The full executable regeneration additionally requires the public versioned code or a reproducible container, regeneration instructions, and a persistent identifier.

## 9. Conclusions

We define BCSD reliability as protocol-conditioned exact-family correctness of a frozen Top-1. Auxiliary views cannot replace it. Cross-view evidence improved discrimination over target score/margin features. GCC was dominated by a strong primary anchor: Full had no statistically significant gain in ROC-AUC over Primary-anchor, whereas Clang benefited from complementary non-primary views. Strong ranking did not ensure stable threshold transfer. On 240 project-identity-disjoint families, the design-locked structural branch accepted 75/240 GCC and 99/240 Clang candidates with no observed family-level errors, without implying zero deployment risk. Correspondence mismatch reduced discrimination toward chance. The corrected TF-IDF remained supportive because repair followed label access. The contribution is a versioned candidate-preserving audit framework with explicit minimum-sufficiency, evidence-status, and deployment boundaries.

## Figures and Tables

**Table 1:** Methodological positioning by endpoint, replacement, calibration, abstention, project isolation, compiler/open-set evidence, and guarantee. The table defines scope, not superiority.

Method	Endpoint	Replace?	Calibration	Abstain	Project Isolation	Compiler/Open-Set Evidence	Guarantee
BCSD [3,4,5,6,7,8,9,10,11,12,13,14,15,16,17,18,19,20,21,22,23,24,25,26]	Pair/rank	Usually	Usually none	Varies	Varies	Compiler/open-set varies	None generally
Calibration [27,28,29]	Event probability	No	Platt/temperature/Dirichlet/isotonic	No	Not intrinsic	Not intrinsic	None
Selective classification [31,32,33,38]	Accept/abstain	No	Optional	Yes	Not intrinsic	Not intrinsic	Method-specific
Conformal/LTT [34,35]	Risk rule/set	No	Not required	Yes	Assumption-dependent	Not intrinsic	Finite-sample under assumptions
Ranked-retrieval risk [37]	Ranked-set risk	Varies	Method-specific	Yes	Method-dependent	Method-specific	Method-specific
This work	Frozen Top-1 correctness	No	Inner out-of-fold (OOF) Platt	Yes	Project-grouped	Same-source; closed-set	No; Wilson diagnostic

**Table 2:** Controlled terminology and principal notation. Each term or symbol has one role throughout this article.

Term/Symbol	Definition	Role
G	Frozen gallery	Gallery
M, s_M	Backend and score	Retrieval
q_i_^0^, ${\hat{g}}_{i}$	Target view; frozen Top-1	Audit object
Y_i_	Exact-family correctness	Endpoint
Z_i_, U_i_, W	Observable state; output; added block	State
X_i_, ΦΛ	Evidence vector; frozen encoder	Features
Πpred, Πdec	Prediction/decision protocol	Scope
p_i_*, ${\hat{p}}_{i}$	Protocol probability; estimate	Probability
V	Auxiliary views	Evidence
𝒮, 𝒞	Project estimation; calibration	Estimation
Aτ,i, Cτ, Rτ	Accept; coverage; risk	Selection
Rfam, Rmacro, Rmax	Family; project-macro; max risk	Risk
τ, α, δ	Threshold; risk budget; upper-tail prob.	Decision
A_−k_, C_−k_	Outer predictor; inner calibrator	Nesting
Candidate-preserving audit	Auxiliary views cannot replace Top-1	Task
Direct	Auxiliary candidate may replace target	Baseline
Primary-anchor	Strongest prespecified auxiliary (GCC-O3)	Evidence
Uniqueness guard	Support plus uniqueness rule	Rule
Full	Calibrated 34-variable logistic auditor	Model

**Table 3:** Frozen evidence blocks defining the candidate-centered auditor.

Block	Dim.	Principal Evidence
Target intrinsic	4	Top-1 score, margin, normalized Top-k entropy, tie multiplicity
Representation ambiguity	1	Byte-equivalence group size of the fixed candidate
Primary-anchor (GCC-O3)	8	Support, reciprocal rank, score, uniqueness, tie, margin, Jaccard@5/@10
Cross-compiler anchor	6	Support, reciprocal rank, score, uniqueness, Jaccard@5/@10
All-auxiliary aggregate	9	Support rate, rank and score summaries, dispersion, overlap, consensus
Primary-excluded aggregate	6	Support, rank, score, and overlap from the remaining views

**Table 4:** Online fixed-candidate reliability auditing.

Step	Operation	Invariant
1	Validate bundle.	Abstain on mismatch.
2	Freeze target Top-1.	No replacement.
3	Extract rank/support/ties/overlap.	No label access.
4	Encode and standardize.	Training semantics.
5	Apply model and Platt map.	Matched bundle.
6	Apply frozen threshold.	No outcome adaptation.

**Table 5:** Nested project-level calibration and threshold freezing.

Stage	Operation	Output/Isolation
Outer	Split projects.	Zero overlap.
Inner OOF	Fit logits and Platt map.	Training-side only.
Threshold	Maximize feasible coverage.	Frozen/reject-all.
Outer score	Refit base; score test.	Held-out risks.
Repeat	Repeat partitions.	Compliance sensitivity.

**Table 6:** Data hierarchy and experimental roles.

Data Level	Families	Projects	Binary Views	Role
Successfully built pool	500	19	3000	Built candidate pool
Primary validation	413	16	2478	Primary validation and thresholds
Clean sensitivity	268	14	1608	Collision/length sensitivity
Challenge intersection	33	–	–	Difficult primary subset
Structural development set	94	–	564 (6/family)	Locks structural backend
Identity-disjoint new projects	240	55	1440	Locked transfer/system comparison

**Table 7:** Evidence-status ledger and permissible inference by analysis.

Analysis Group	Purpose	Evidence Role	Scope of Inference
Freeze/anchor	Freeze/anchor	Mechanism	Anchor diagnosis
Guard/collisions	Guard/collisions	Boundary	False consensus, ambiguity
Construction	Build/eligibility	Construction	Qualified cohort
Reliability	Calibration	Internal validation	Discrimination, calibration
Thresholds	Thresholds	Operating point	Held-out operating-point stability
Structural backend	Backend tests	Locked evaluation	Rank versus scale
Identity-disjoint	New projects	Protocol-matched transfer	Project-identity transfer
Mismatch/systems	Mismatch/systems	Post hoc boundary	Correspondence/sufficiency

**Table 8:** Internal TF-IDF retrieval baselines for the 413-family primary-validation cohort.

Query View	Exact R@1	Byte-Equivalent R@1	R@5	R@10	Role
GCC-O0 target	0.5061	0.5278	0.7070	0.7676	Audited target
Clang-O0 target	0.4310	0.4479	0.6489	0.7070	Audited target
GCC-O3 anchor	0.9709	0.9976	0.9976	0.9976	Dominant auxiliary
Clang-O2 auxiliary	0.8571	0.8814	0.9467	0.9613	Clang same-toolchain evidence
Clang-O3 auxiliary	0.8499	0.8741	0.9467	0.9613	Additional Clang evidence

**Table 9:** Internal project-OOF learned models and deterministic minimum-sufficiency rules.

Task	Model	Type	ROC-AUC	Brier	AURC	Accepted/Errors (Coverage)
GCC-O0	Primary-support rule	Rule	0.9779	0.0218	—	218/9 (52.8%)
GCC-O0	Uniqueness Guard	Rule	0.9713	0.0291	—	197/0 (47.7%)
GCC-O0	Score + Margin	Learned	0.8343	0.1667	0.2428	—
GCC-O0	Primary-anchor	Learned	0.9994	0.0088	0.1496	—
GCC-O0	Multi-aux-no-primary	Learned	0.9891	0.0395	0.1548	—
GCC-O0	Full	Learned	0.9995	0.0085	0.1495	—
Clang-O0	Primary-support rule	Rule	0.9303	0.0654	—	169/9 (40.9%)
Clang-O0	Uniqueness Guard	Rule	0.9199	0.0702	—	153/2 (37.0%)
Clang-O0	Score + Margin	Learned	0.7926	0.1801	0.3264	—
Clang-O0	Primary-anchor	Learned	0.9858	0.0343	0.2176	—
Clang-O0	Multi-aux-no-primary	Learned	0.9991	0.0113	0.2067	—
Clang-O0	Full	Learned	0.9995	0.0084	0.2065	—

**Table 10:** Paired comparisons between project-cluster increments of Full and prespecified minimum-sufficiency.

Task	Comparator	ΔROC-AUC (95% CI)	ΔAURC (95% CI)
GCC-O0	Score + Margin	+0.1655 [0.1335, 0.2010]	−0.0921 [−0.1137, −0.0665]
GCC-O0	Primary-anchor	+0.0002 [−0.0002, 0.0007]	−0.0001 [−0.0004, 0.0001]
GCC-O0	Multi-aux-no-primary	+0.0103 [0.0026, 0.0199]	−0.0052 [−0.0098, −0.0014]
Clang-O0	Score + Margin	+0.2079 [0.1608, 0.2534]	−0.1209 [−0.1597, −0.0888]
Clang-O0	Primary-anchor	+0.0139 [0.0051, 0.0241]	−0.0108 [−0.0193, −0.0027]
Clang-O0	Multi-aux-no-primary	+0.0004 [−0.0001, 0.0011]	−0.0002 [−0.0006, 0.0001]

**Table 11:** Frozen-threshold results over 10 held-out-project repetitions. Wilson compliance is diagnostics only.

Task	Model	Budget	Mean Coverage	Mean Empirical Risk	Repetitions Meeting Criterion
GCC-O0	Full	5%	0.5102	0.0216	5/10
GCC-O0	Primary-anchor	5%	0.5099	0.0193	7/10
GCC-O0	Full	10%	0.5426	0.0674	7/10
GCC-O0	Primary-anchor	10%	0.5412	0.0649	10/10
Clang-O0	Full	5%	0.3952	0.0095	10/10
Clang-O0	Multi-aux-no-primary	5%	0.3855	0.0069	10/10
Clang-O0	Full	10%	0.4533	0.0517	10/10

**Table 12:** Structural internal/identity-disjoint results plus the original and corrected TF-IDF transfer. Structural rows use the design-locked backend. Table 9 uses TF-IDF. Corrected TF-IDF is supportive only.

Evidence Level	Metric	GCC-O0	Clang-O0
Internal (413; structural)	Structural target R@1	0.2131	0.2978
Internal (413; structural)	Full mean ROC-AUC	0.9976	0.9974
Internal (413; structural)	5% held-out Wilson compliance	Primary-anchor 10/10	Full 4/10
Transfer (240; structural)	Structural target R@1	0.3125	0.4333
Transfer (240; structural)	Selected-model ROC-AUC	Primary-anchor 1.0000	Full 1.0000
Transfer (240; structural)	Accepted/errors	75/0	99/0
Transfer (240; structural)	Coverage	0.3125	0.4125
Transfer (240; structural)	Wilson upper bound	0.0348	0.0266
Original locked TF-IDF	Accepted/errors	182/135	240/142
Original locked TF-IDF	Empirical risk/Wilson bound	0.7418/0.7913	0.5917/0.6426
Corrected TF-IDF (supportive)	Accepted/errors	47/0	98/0
Corrected TF-IDF (supportive)	Coverage/Wilson bound	0.1958/0.0544	0.4083/0.0269

**Table 13:** Post hoc correspondence mismatch with candidates and labels fixed.

Backend	Task	True ROC-AUC	Mismatch ROC-AUC	Mismatch Accepted/Errors	Mismatch Risk
TF-IDF	GCC-O0	1.0000	0.4363	47/40	0.8511
TF-IDF	Clang-O0	1.0000	0.5211	98/62	0.6327
Structural	GCC-O0	1.0000	0.4695	75/56	0.7467
Structural	Clang-O0	1.0000	0.5174	100/55	0.5500

**Table 14:** Principal validity threats, controls, and unresolved scope limitations.

Dimension	Implemented Control	Remaining Limitation
Construct	Frozen exact-family label; byte equivalence separate	Identity ≠ semantic equivalence
Internal	Candidate/label isolation; nested projects; mismatch	Residual implementation/confounding
Statistical	Repeated project splits; cluster sensitivity; hierarchical risks	16 projects; Wilson diagnostic; no alternative-calibrator/formal-risk comparison
External	Locked structural backend; 55 new projects without project/hash overlap	One ecosystem; x86-64; closed set; complete views; no neural/shift/obfuscation validation
Reproducibility	Versioned manifests, commands, environments, hashes, predictions, thresholds	Executable regeneration is outside the current public release
Computation	Retrieval-call burden stated	No wall-clock/storage/energy benchmark
Governance	Design locks; original failure retained; evidence roles separated	Not preregistered; corrected TF-IDF lacks second untouched confirmation; post hoc analyses are nonconfirmatory

## Data Availability

The source benchmark is AnghaBench [40]. Supplementary File S1 and Supplementary Data S1 accompany this article and provide the evidence ledger, candidate-level diagnostics, operating-point summaries, bootstrap summaries, split audits, and archive manifest used to audit the reported analyses. These materials support independent inspection of the reported results. Independently executable end-to-end regeneration would additionally require the original analysis code and matched computational environment, which are not included in the accompanying supplementary package.

## Supplementary Materials

The following supporting information can be downloaded at: https://github.com/anyiming01/Paper-Supplementary-Materials, accessed on 18 August 2026. Supplementary File S1: representative candidate trajectories illustrating stable support, false consensus, byte collision, and cross-view conflict; Table S1: project-level correct/error counts and calibration summaries for Full on the 413-family primary-validation cohort; Table S2: repeated-project operating-point summaries under one-sided Wilson screening; Table S3: outer-fold threshold ranges and repetition-level held-out outcomes for the representative 5% Wilson operating points; Table S4: project-split audit summary for the representative nested operating-point evaluations; Figure S1: project-level calibration for Full; Figure S2: global reliability diagram for Full using the archived calibration-bin summaries; Figure S3: tie-aware accepted-risk-versus-coverage curves for Full and Primary-anchor on the primary-validation cohort; Figure S4: hierarchical accepted-set risk at the representative 5% Wilson operating points across repeated project partitions.

## References

[1] Haq I.U., Caballero J. (2021). A survey of binary code similarity. ACM Comput. Surv..

[2] Du J., Wei Q., Wang Y., Sun X. (2023). A review of deep learning-based binary code similarity analysis. Electronics.

[3] Yang S., Xu Z., Xiao Y., Lang Z., Tang W., Liu Y., Shi Z., Li H., Sun L. (2023). Towards practical binary code similarity detection: Vulnerability verification via patch semantic analysis. ACM Trans. Softw. Eng. Methodol..

[4] PPewny J., Garmany B., Gawlik R., Rossow C., Holz T. Cross-architecture bug search in binary executables. Proceedings of the 2015 IEEE Symposium on Security and Privacy.

[5] Eschweiler S., Yakdan K., Gerhards-Padilla E. discovRE: Efficient cross-architecture identification of bugs in binary code. Proceedings of the Network and Distributed System Security (NDSS) Symposium.

[6] Yamaguchi F., Golde N., Arp D., Rieck K. Modeling and discovering vulnerabilities with code property graphs. Proceedings of the 2014 IEEE Symposium on Security and Privacy.

[7] Feng Q., Zhou R., Xu C., Cheng Y., Testa B., Yin H. Scalable graph-based bug search for firmware images. Proceedings of the 2016 ACM SIGSAC Conference on Computer and Communications Security.

[8] Xu X., Liu C., Feng Q., Yin H., Song L., Song D. Neural network-based graph embedding for cross-platform binary code similarity detection. Proceedings of the 2017 ACM SIGSAC Conference on Computer and Communications Security.

[9] Liu B., Huo W., Zhang C., Li W., Li F., Piao A., Zou W. αDiff: Cross-version binary code similarity detection with DNN. Proceedings of the 33rd ACM/IEEE International Conference on Automated Software Engineering.

[10] Massarelli L., Di Luna G.A., Petroni F., Baldoni R., Querzoni L. SAFE: Self-attentive function embeddings for binary similarity. Proceedings of the 16th International Conference, DIMVA 2019.

[11] Ding S.H.H., Fung B.C.M., Charland P. Asm2Vec: Boosting static representation robustness for binary clone search against code obfuscation and compiler optimization. Proceedings of the 2019 IEEE Symposium on Security and Privacy (SP).

[12] Duan Y., Li X., Wang J., Yin H. DeepBinDiff: Learning program-wide code representations for binary diffing. Proceedings of the Network and Distributed System Security (NDSS) Symposium.

[13] Yu Z., Cao R., Tang Q., Nie S., Huang J., Wu S. Order matters: Semantic-aware neural networks for binary code similarity detection. Proceedings of the AAAI Conference on Artificial Intelligence.

[14] Li X., Qu Y., Yin H. PalmTree: Learning an assembly language model for instruction embedding. Proceedings of the 2021 ACM SIGSAC Conference on Computer and Communications Security.

[15] Yang J., Fu C., Liu X.-Y., Yin H., Zhou P. (2022). Codee: A tensor embedding scheme for binary code search. IEEE Trans. Softw. Eng..

[16] Yang S., Cheng L., Zeng Y., Lang Z., Zhu H., Shi Z. Asteria: Deep learning-based AST-encoding for cross-platform binary code similarity detection. Proceedings of the 2021 51st Annual IEEE/IFIP International Conference on Dependable Systems and Networks (DSN).

[17] Wang H., Qu W., Katz G., Zhu W., Gao Z., Qiu H., Zhuge J., Zhang C. jTrans: Jump-aware transformer for binary code similarity detection. Proceedings of the 31st ACM SIGSOFT International Symposium on Software Testing and Analysis.

[18] Marcelli A., Graziano M., Ugarte-Pedrero X., Fratantonio Y. How machine learning is solving the binary function similarity problem: A large-scale evaluation. Proceedings of the USENIX Security.

[19] Jiang S., Fu C., Qian Y., He S., Lv J., Han L. (2022). IFAttn: Binary code similarity analysis based on interpretable features with attention. Comput. Secur..

[20] Jiang S., Fu C., He S., Lv J., Han L., Hu H. (2024). BinCola: Diversity-sensitive contrastive learning for binary code similarity detection. EEE Trans. Softw. Eng..

[21] Wang H., Ma P., Wang S., Tang Q., Nie S., Wu S. (2023). sem2vec: Semantics-aware assembly tracelet embedding. ACM Trans. Softw. Eng. Methodol..

[22] Wang H., Ma P., Yuan Y., Liu Z., Wang S., Tang Q., Nie S., Wu S. (2023). Enhancing DNN-based binary code function search with low-cost equivalence checking. IEEE Trans. Softw. Eng..

[23] He H., Lin X., Weng Z., Zhao R., Gan S., Chen L., Ji Y., Wang J., Xue Z. Code is not natural language: Unlock the power of semantics-oriented graph representation for binary code similarity detection. Proceedings of the USENIX Security Symposium.

[24] Wang J., Zhang C., Chen L., Rong Y., Wu Y., Wang H., Tan W., Li Q., Li Z. Improving ML-based binary function similarity detection by assessing and deprioritizing control flow graph features. Proceedings of the USENIX Security Symposium.

[25] Ren T., Che R., Gilman G.R., De Carli L., Walls R.J. REVDECODE: Enhancing binary function matching with context-aware graph representations and relevance decoding. Proceedings of the USENIX Security Symposium.

[26] Gu Y., Shu H., Kang F., Hu F. (2025). UniASM: Binary code similarity detection without fine-tuning. Neurocomputing.

[27] Platt J.C. (2000). Probabilistic outputs for support vector machines and comparisons to regularized likelihood methods. Advances in Large Margin Classifiers.

[28] Guo C., Pleiss G., Sun Y., Weinberger K.Q. On calibration of modern neural networks. Proceedings of the 34th International Conference on Machine Learning.

[29] Kull M., Perello-Nieto M., Kängsepp M., Filho T.S., Song H., Flach P. Beyond temperature scaling: Obtaining well-calibrated multiclass probabilities with Dirichlet calibration. Proceedings of the 33rd International Conference on Neural Information Processing Systems.

[30] Brier G.W. (1950). Verification of forecasts expressed in terms of probability. Mon. Weather. Rev..

[31] Geifman Y., El-Yaniv R. Selective classification for deep neural networks. Proceedings of the Neural Information Processing Systems.

[32] Geifman Y., El-Yaniv R. SelectiveNet: A deep neural network with an integrated reject option. Proceedings of the 36th International Conference on Machine Learning.

[33] El-Yaniv R., Wiener Y. (2010). On the foundations of noise-free selective classification. J. Mach. Learn. Res..

[34] Angelopoulos A.N., Bates S., Fisch A., Lei L., Schuster T. Conformal risk control. Proceedings of the International Conference on Learning Representations 2024.

[35] Angelopoulos A.N., Bates S., Candès E.J., Jordan M.I., Lei L. (2025). Learn then test: Calibrating predictive algorithms to achieve risk control. Ann. Appl. Stat..

[36] Liang H., Peng L., Sun J. (2024). Selective classification under distribution shifts. Trans. Mach. Learn. Res..

[37] Xu Y., Ying M., Guo W., Wei Z. Two-stage risk control with application to ranked retrieval. Proceedings of the Thirty-Fourth International Joint Conference on Artificial Intelligence.

[38] da Silva A.F., Kind B.C., Magalhaes J.W.d.S., Rocha J.N., Guimaraes B.C.F., Pereira F.M.Q. AnghaBench: A suite with one million compilable C benchmarks for code-size reduction. Proceedings of the 2021 IEEE/ACM International Symposium on Code Generation and Optimization (CGO).

[39] Wilson E.B. (1927). Probable inference, the law of succession, and statistical inference. J. Am. Stat. Assoc..

[40] Efron B. (1979). Bootstrap methods: Another look at the jackknife. Ann. Stat..

